# Intronic sequences are required for *AINTEGUMENTA*-*LIKE6* expression in *Arabidopsis* flowers

**DOI:** 10.1186/s13104-015-1537-6

**Published:** 2015-10-12

**Authors:** Beth A. Krizek

**Affiliations:** Department of Biological Sciences, University of South Carolina, Columbia, SC 29208 USA

**Keywords:** *AINTEGUMENTA*-*LIKE6* (*AIL6*), *AINTEGUMENTA*-*LIKE/PLETHORA* (*AIL/PLT*) family, *AINTEGUMENTA* (*ANT*), Flower development, *Arabidopsis thaliana*, Transcription factors, Enhancers, Gene regulation

## Abstract

**Background:**

The *AINTEGUMENTA*-*LIKE6/PLETHORA3* (*AIL6/PLT3*) gene of *Arabidopsis thaliana* is a key regulator of growth and patterning in both shoots and roots. *AIL6* encodes an AINTEGUMENTA-LIKE/PLETHORA (AIL/PLT) transcription factor that is expressed in the root stem cell niche, the peripheral region of the shoot apical meristem and young lateral organ primordia. In flowers, *AIL6* acts redundantly with *AINTEGUMENTA* (*ANT*) to regulate floral organ positioning, growth, identity and patterning. Experiments were undertaken to define the genomic regions required for *AIL6* function and expression in flowers.

**Results:**

Transgenic plants expressing a copy of the coding region of *AIL6* in the context of 7.7 kb of 5′ sequence and 919 bp of 3′ sequence (*AIL6:cAIL6*-*3′*) fail to fully complement *AIL6* function when assayed in the *ant*-*4 ail6*-*2* double mutant background. In contrast, a genomic copy of *AIL6* with the same amount of 5′ and 3′ sequence (*AIL6:gAIL6*-*3′*) can fully complement *ant*-*4 ail6*-*2*. In addition, a genomic copy of *AIL6* with 590 bp of 5′ sequence and 919 bp of 3′ sequence (*AIL6m:gAIL6*-*3′*) complements *ant*-*4 ail6*-*2* and contains all regulatory elements needed to confer normal *AIL6* expression in inflorescences. Efforts to map cis-regulatory elements reveal that the third intron of *AIL6* contains enhancer elements that confer expression in young flowers but in a broader pattern than that of *AIL6* mRNA in wild-type flowers. Some *AIL6:gAIL6*-*3′* and *AIL6m:gAIL6*-*3′* lines confer an over-rescue phenotype in the *ant*-*4 ail6*-*2* background that is correlated with higher levels of *AIL6* mRNA accumulation.

**Conclusions:**

The results presented here indicate that *AIL6* intronic sequences serve as transcriptional enhancer elements. In addition, the results show that increased expression of *AIL6* can partially compensate for loss of *ANT* function in flowers.

## Background

*AIL6* encodes a member of the small subfamily of AIL/PLT transcription factors that are part of the large AP2/ERF family in *Arabidopsis thaliana* [1]. AIL proteins are key regulators of developmental processes throughout the plant life cycle (reviewed in [2]). *AIL6* regulates multiple processes during Arabidopsis root and shoot development, largely in a redundant fashion with other *AIL* genes. Loss of *AIL6* function on its own has no obvious phenotype in the shoot and only results in a slightly shorter root and root apical meristem [3, 4]. Within shoots, *AIL6* acts with *ANT* and *AINTEGUMENTA*-*LIKE7/PLETHORA7* (*AIL7/PLT7*) to maintain the shoot apical meristem during vegetative development and works in a redundant fashion with *AINTEGUMENTA*-*LIKE5/PLETHORA5* (*AIL5/PLT5*) and *AIL7* to control shoot phyllotaxy [5, 6]. In flowers, *AIL6* acts redundantly with *ANT* to regulate floral organ initiation, growth, identity specification and patterning [4]. *AIL6* function is required for root formation in combination with *PLETHORA1* (*PLT1*) and *PLETHORA2* (*PLT2*) and controls the positioning of lateral roots in a redundant fashion with *AIL5* and *AIL7* [3, 7].

As expected from its functions throughout the plant, *AIL6* is expressed at the mRNA level in multiple tissues. In roots, *AIL6* mRNA is detected in the stem cell niche, pericycle cells prior to lateral root initiation, and lateral root primordia [3, 7]. In the shoot, *AIL6* mRNA is detected in young lateral organ primordia and throughout the shoot apical meristem with expression higher in the periphery of the meristem and incipient lateral organ as compared with the center of the meristem [1, 5]. In flowers, *AIL6* expression is associated with young flower primordium and early stages of floral organ development. *AIL6* is expressed throughout stage one and two flower primordia, becoming primarily restricted to the floral meristem dome in stage three flowers with only low levels present in sepal primordia [1]. In stage six flowers, some *AIL6* mRNA is present within petal, stamen and carpel primordia; only low amounts of *AIL6* mRNA are detected after this stage of development [1].

In several of these tissues, *AIL6* expression is linked with the activity of AUXIN RESPONSE FACTORs (ARFs), transcription factors that regulate gene expression in response to auxin. In lateral roots, *AIL6* appears to act downstream of *ARF7* and *ARF19* [7]. *AIL6* is not expressed in any pericycle cells of *arf7 arf19* double mutants, which lack most lateral roots, although it is not known whether *AIL6* is a direct target of these transcription factors [7]. Chromatin immunoprecipitation (ChIP) experiments show that MONOPTEROS/AUXIN RESPONSE FACTOR 5 (MP/ARF5) directly activates *AIL6* in the periphery of the shoot apical meristem to promote flower primordium initiation [8]. Two other potential regulators of *AIL6* are the floral meristem identity protein LEAFY (LFY) and APETALA1 (AP1) which were shown to bind to *AIL6* in genome-wide ChIP experiments [9, 10].

Despite the importance of *AIL6* in Arabidopsis vegetative and reproductive development, sequences required for proper *AIL6* expression have not been identified. Here I define the genomic regions necessary for *AIL6* function in flowers by complementation of *ant*-*4 ail6*-*2* double mutants with transgenes containing different amounts of *AIL6* sequence. These experiments show that introns are required for *AIL6* function and expression in flowers. In particular, intron three was found to contain enhancer elements that drive *AIL6* expression in early stages of flower development. The importance of intron three to *AIL6* regulation is also demonstrated by work from other labs showing binding of LFY and AP1 to the third intron of *AIL6* [9, 10]. Furthermore, this work demonstrates that increased expression of *AIL6* can partially compensate for loss of *ANT* function.

## Results and discussion

### Intronic sequences are required for complementation of *AIL6* function in *ant ail6* flowers

**Fig. 1:**
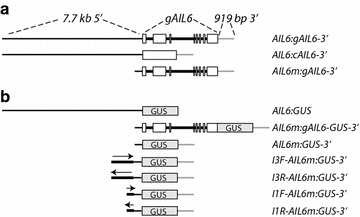
*AIL6* constructs used in this study. **a** Constructs tested for complementation of *AIL6* function in the *ant*-*4 ail6*-*2* double mutant background. *AIL6* 5′ sequences are shown with *thin black lines*, exons with *white boxes*, introns with *thick black lines*, and *AIL6* 3′ sequences with *gray lines*. *cAIL6* corresponds to the coding region of *AIL6*. *AIL6m* corresponds to 590 bp of 5′ sequence directly upstream of the start codon. **b** GUS reporter constructs. *AIL6* 5′ sequences are shown with *thin black lines*, exons with *white boxes*, introns with *thick black lines*, and *AIL6* 3′ sequences with *gray lines*. *Arrows* show the direction (Forward, *F*; or Reverse, *R*) for introns three (I3) and one (I1) in the reporter constructs

**Fig. 2:**
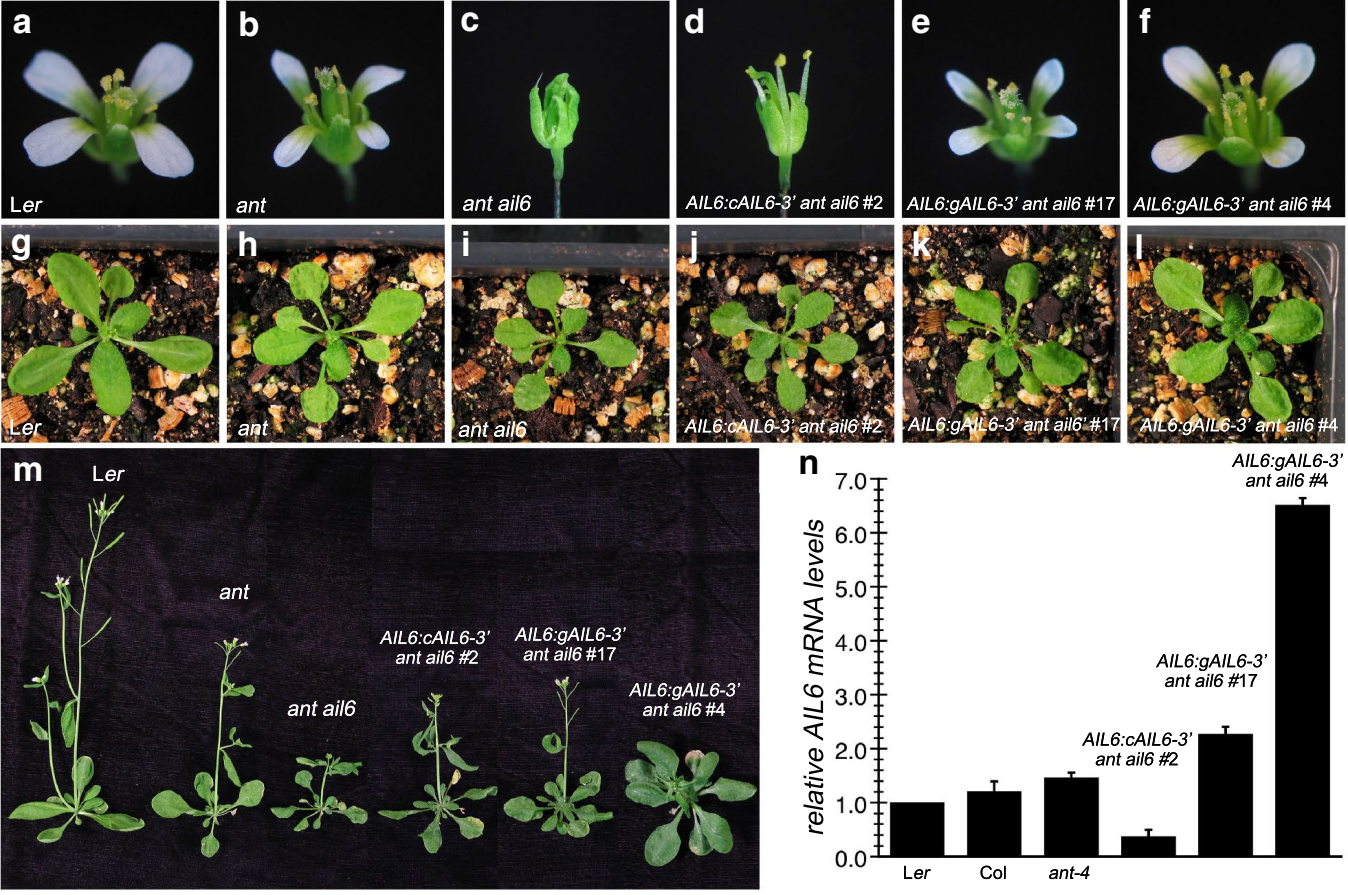
**a** Genomic copy of *AIL6* with 7.7 kb of 5′ and 919 of 3′ sequence rescues *ant ail6*. Flowers from L*er* (**a**), *ant*-*4* (**b**), *ant*-*4 ail6*-*2* (**c**), *AIL6:cAIL6*-*3′ ant ail6* line 2 (**d**), *AIL6:gAIL6*-*3′ ant ail6* line 17 (**e**), *AIL6:gAIL6*-*3′ ant ail6* line 4 (**f**). 20 day old plants corresponding to the following genotypes: L*er* (**g**), *ant*-*4* (**h**), *ant*-*4 ail6*-*2* (**i**), *AIL6:cAIL6*-*3′ ant ail6* line 2 (**j**), *AIL6:gAIL6*-*3′ ant ail6* line 17 (**k**), *AIL6:gAIL6*-*3′ ant ail6* line 4 (**l**). **m** 30 day old plants of the indicated genotypes. **n** Graph of RT-qPCR results showing relative *AIL6* mRNA levels in L*er*, Col, *ant*-*4*, *AIL6:cAIL6*-*3′ ant ail6* line 2 and *AIL6:gAIL6*-*3′ ant ail6* lines 17 and 4. Expression in L*er* is set to one and the *error bars* show standard deviation

To define the genomic regions required for *AIL6* function in flower development, *ant*-*4/*+ *ail6*-*2* plants were transformed with a transgene containing the *AIL6* coding region in a genomic context of 7.7 kb of 5′ sequence and 919 bp of 3′ sequence (i.e. *AIL6:cAIL6*-*3′*; Fig. 1a). The *ant*-*4 ail6*-*2* double mutant was used in these studies since *ail6*-*2* single mutants show no flower phenotype [4]. Of the six transgenic lines obtained, three lines (lines 2, 9, and 10) showed a partial rescue of the *ant*-*4 ail6*-*2* double mutant phenotype while three lines (lines 11, 14 and 16) exhibited no rescue. In the partially rescued *AIL6:cAIL6*-*3′ ant*-*4 ail6*-*2* line 2, the flowers are intermediate in severity between those of *ant*-*4* and those of *ant*-*4 ail6*-*2* (Fig. 2a–d). They are larger than those of *ant*-*4 ail6*-*2* and consist primarily of sepals, filaments, stamens and carpel valves fused to varying extents (Fig. 2d; Table 1). In contrast, *ant*-*4 ail6*-*2* flowers consist of sepals, filaments, stamenoid organs and unfused carpel valves (Fig. 2c; Table 1). The stamens in *AIL6:cAIL6*-*3′ ant*-*4 ail6*-*2* line 2 flowers resemble those of *ant*-*4* with two locules rather than the four locules present in wild-type stamens, and some produce pollen. No petals were produced in these flowers. The leaves of *AIL6:cAIL6*-*3′ ant*-*4 ail6*-*2* line 2 were similar in size to those of *ant*-*4 ail6*-*2* in 20 day old plants (Fig. 2g–j).

**Table 1:** Floral organ counts of genotypes utilized in this study

	L*er*	*ant*-*4*	*ant*-*4 ail6*-*2*	*AIL6:cAIL6*-*3′* line 2	*AIL6:gAIL6*-*3′* line 17	*AIL6:gAIL6*-*3′* line 4	*AIL6m:gAIL6*-*3′* line 46	*AIL6m:gAIL6*-*3′* line 31
Se	4.0	4.01	4.23	4.03	4.0	4.0	4.0	4.0
Pe/Se		0.01		0.01				
Other flat green			0.14	0.05				
Pe	4.02	3.78			3.77	3.30	3.97	3.99
Pe/St	0.01	0.01						
fil		0.03	0.45	0.78	0.05	0.02	0.01	0.02
Flat St-like			0.22	0.09				
St-like fil			0.29	0.84				
St	5.90	4.44	0.07	2.48	4.5	5.07	4.88	5.56
St-like valve			0.15	0.03				
Ca valve			1.75	1.40				
Ca	2.0	2.0			1.99	2.0	2.0	2.0
Central fil			0.23	0.08				

*Se* sepal, *Pe*/*Se* petaloid sepal, *Pe* petal, *Pe*/*St* petaloid stamen, *St* stamen, *Ca* carpel, *fil* filament

To determine whether the lack of complementation by the *AIL6:cAIL6*-*3′* transgene was a consequence of the absence of introns or insufficient 5′ and 3′ sequence, *ant*-*4/*+ *ail6*-*2* plants were transformed with a transgene corresponding to a genomic copy of *AIL6* in the same context of 5′ and 3′ sequence (*AIL6:gAIL6*-*3′*) (Fig. 1a). Of the 5 lines obtained, one line (line 12) exhibited partial rescue of *ant*-*4ail6*-*2*, two lines (lines 11 and 17) exhibited complete rescue such that the flowers resembled *ant*-*4*, and two lines (lines 4 and 21) exhibited an over-rescue phenotype such that the plants had a less severe phenotype than *ant*-*4* (Fig. 2e, f). The phenotypic variation in the degree of complementation in these lines is presumably a consequence of variation in transgene insertion sites. *AIL6:gAIL6*-*3′ant*-*4ail6*-*2* line 17 flowers closely resemble *ant*-*4* flowers with regard to the identity, numbers, and size of the floral organs (Fig. 2e; Table 1). In flowers of the over-rescue *AIL6:gAIL6*-*3′ ant*-*4 ail6*-*2* line 4, the petals are larger than those of *ant*-*4* and the stamen anthers consist of four locules (Fig. 2f). However, *AIL6:gAIL6*-*4′ ant*-*4 ail6*-*2* line 4 petals are not as big as those of wild-type flowers, and the flowers are female sterile and consist of fewer petals and stamens compared to wild-type (Fig. 2f; Table 1). Similar effects were observed with regard to complementation of leaf growth defects in *AIL6:gAIL6*-*3′ ant*-*4 ail6*-*2* lines 17 and 4; line 17 leaves resemble those of *ant*-*4* while line 4 leaves are larger (Fig. 2k, l, m).

### *AIL6* mRNA accumulation depends on intronic sequences

To determine whether the distinct phenotypes conferred by the *AIL6:cAIL6*-*3′* and *AIL6:gAIL6*-*3′* transgenes correlated with the levels of *AIL6* mRNA accumulation in these plants, *AIL6* mRNA levels were examined by reverse transcriptase quantitative PCR (RT-qPCR). *AIL6:cAIL6*-*3′ ant*-*4 ail6*-*2* line 2 inflorescences accumulate less *AIL6* mRNA compared with L*er* (Fig. 2n). *AIL6:gAIL6*-*3′ ant*-*4 ail6*-*2* lines 17 and 4 accumulate more *AIL6* mRNA than L*er*, approximately 2.3 and 6.5 fold higher levels than L*er*, respectively (Fig. 2n). Since the *ant*-*4 ail6*-*2* double mutant is in a mixed Ler/Col background (see “Methods”), I also examined *AIL6* mRNA levels in Col and found them to be similar to those in L*er* (Fig. 2n). *AIL6* mRNA levels were slightly increased in the *ant*-*4* background, suggesting possible cross-regulation of *AIL6* expression. The increased levels of *AIL6* mRNA in lines 17 and 4 compared to L*er*, Col and *ant*-*4* is likely a consequence of the chromosomal position of the transgene insertion site. The RT-qPCR results indicate that intronic sequences increase steady-state *AIL6* mRNA levels. They also suggest that the inability of *AIL6:cAIL6*-*3′* to fully complement *AIL6* function in the *ant*-*4 ail6*-*2* double mutant results from insufficient *AIL6* mRNA. Furthermore, the higher *AIL6* mRNA levels in *AIL6:gAIL6*-*3′ ant*-*4 ail6*-*2* line 4 as compared with line 17 suggest that increased *AIL6* activity can partially compensate for loss of *ANT* function.

**Fig. 3:**
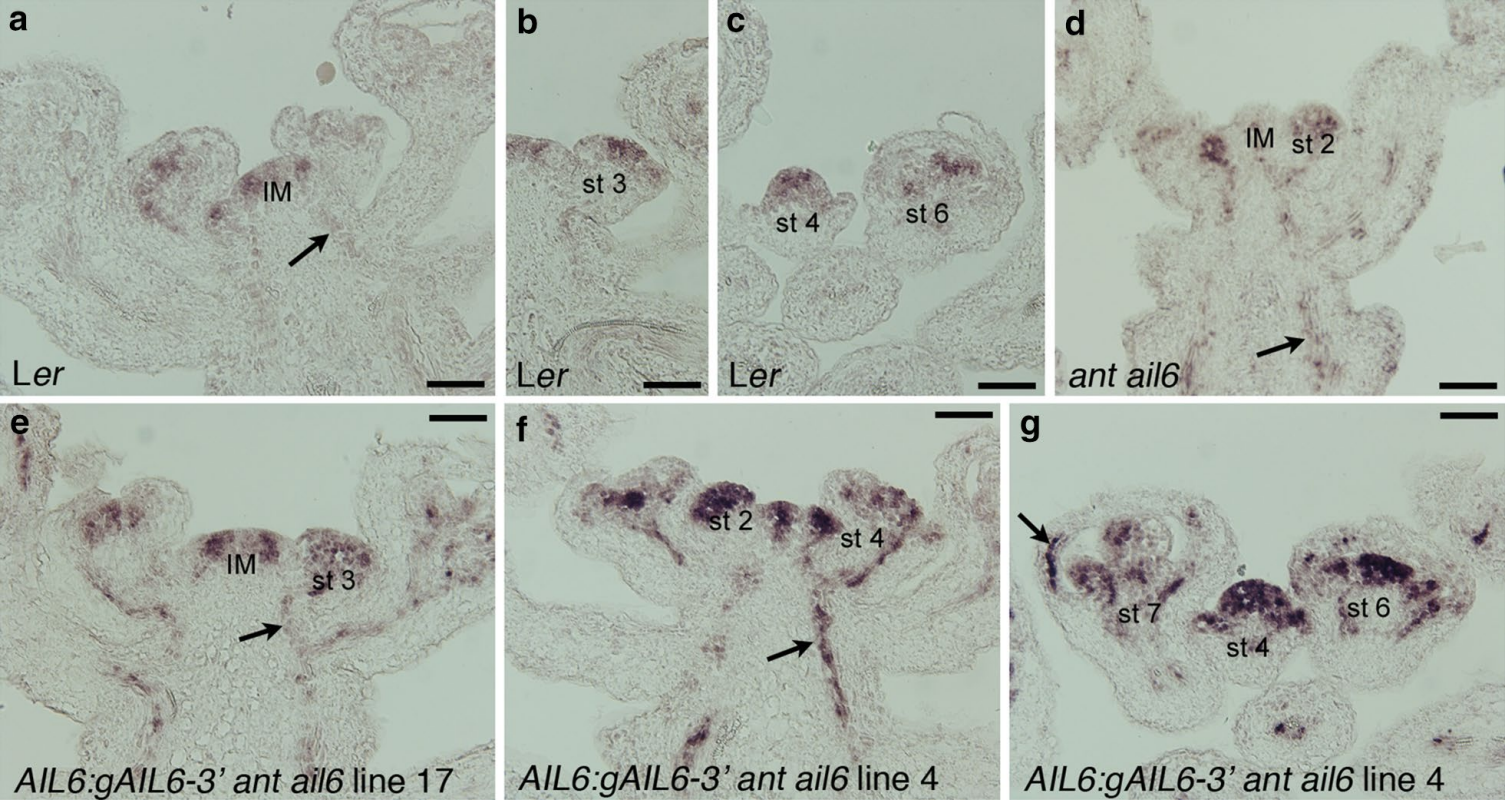
In situ hybridization of *AIL6* mRNA expression in *AIL6:gAIL6*-*3′ ant ail6*. *AIL6* expression in L*er* inflorescence (**a**), L*er* stage 3 flower (**b**), L*er* stage 4 and 6 flowers (**c**), *ant*-*4 ail6*-*2* inflorescence (**d**), *AIL6:gAIL6* -*3′ ant ail6* *line* 17 inflorescence, and *AIL6:gAIL6*-*3′ ant ail6* *line* 4 inflorescences (**e**, **f**). *Arrows* point to staining in the procambium in **a**, **d**–**f** and in a developing sepal in **g**. *Size bars* correspond to 50 μm. The images in **a**–**c** and **e**–**g** are from slides in the same experiment and exposed to substrate for the same length of time. The image in **d** is from a different experiment. *IM* inflorescence meristem, *st 2* stage 2 flower, *st 3* stage 3 flower, *st 4* stage 4 flower, *st 6* stage 6 flower and *st 7* stage 7 flower

The spatial distribution of *AIL6* mRNA in L*er* and *AIL6:gAIL6*-*3′ ant*-*4 ail6*-*2* lines 17 and 4 was examined by in situ hybridization. Within the inflorescence meristem, *AIL6* mRNA accumulates in a similar spatial pattern in L*er* and *AIL6:gAIL6*-*3′ ant*-*4 ail6*-*2* lines 17 and 4, with expression higher in the periphery of the meristem compared with the center (Fig. 3a, e). *AIL6* mRNA accumulates throughout stage 1 and 2 flower primordia on the flanks of the inflorescence meristem in both L*er* and *AIL6:gAIL6*-*3′ ant*-*4 ail6*-*2* lines 17 and 4 (Fig. 3a, f). *AIL6* mRNA was detectable within sepal primordia of *AIL6:gAIL6*-*3′ ant*-*4 ail6*-*2* line 4 stage 4, 6 and 7 flowers, while sepals of stage 4 and 6 L*er* flowers have no detectable *AIL6* mRNA (Fig. 3b, c, g). Little *AIL6* mRNA is detected in any floral organ after stage 6 of flower development (Fig. 3a–c). *AIL6* was expressed in the procambium in *AIL6:gAIL6*-*3′ ant*-*4 ail6*-*2* lines 17 and 4 at noticeable higher levels than in L*er* (Fig. 3a, e–g). This may in part be due to the *ant*-*4 ail6*-*2* background, as *AIL6* mRNA was also more easily detected in the procambium of *ant*-*4 ail6*-*2* double mutants compared to L*er* (Fig. 3d). In summary, the *AIL6* mRNA expression pattern in *AIL6:gAIL6*-*3′ ant*-*4 ail6*-*2* line 17 flowers closely resembles that of L*er*, while *AIL6* mRNA is detected in a broader pattern in *AIL6:gAIL6*-*3 ant*-*4 ail6*-*2* line 4 stage 4 flowers and persists longer in developing floral organs.

### Complementation of *AIL6* function in *ant ail6* flowers by a smaller genomic fragment

**Fig. 4:**
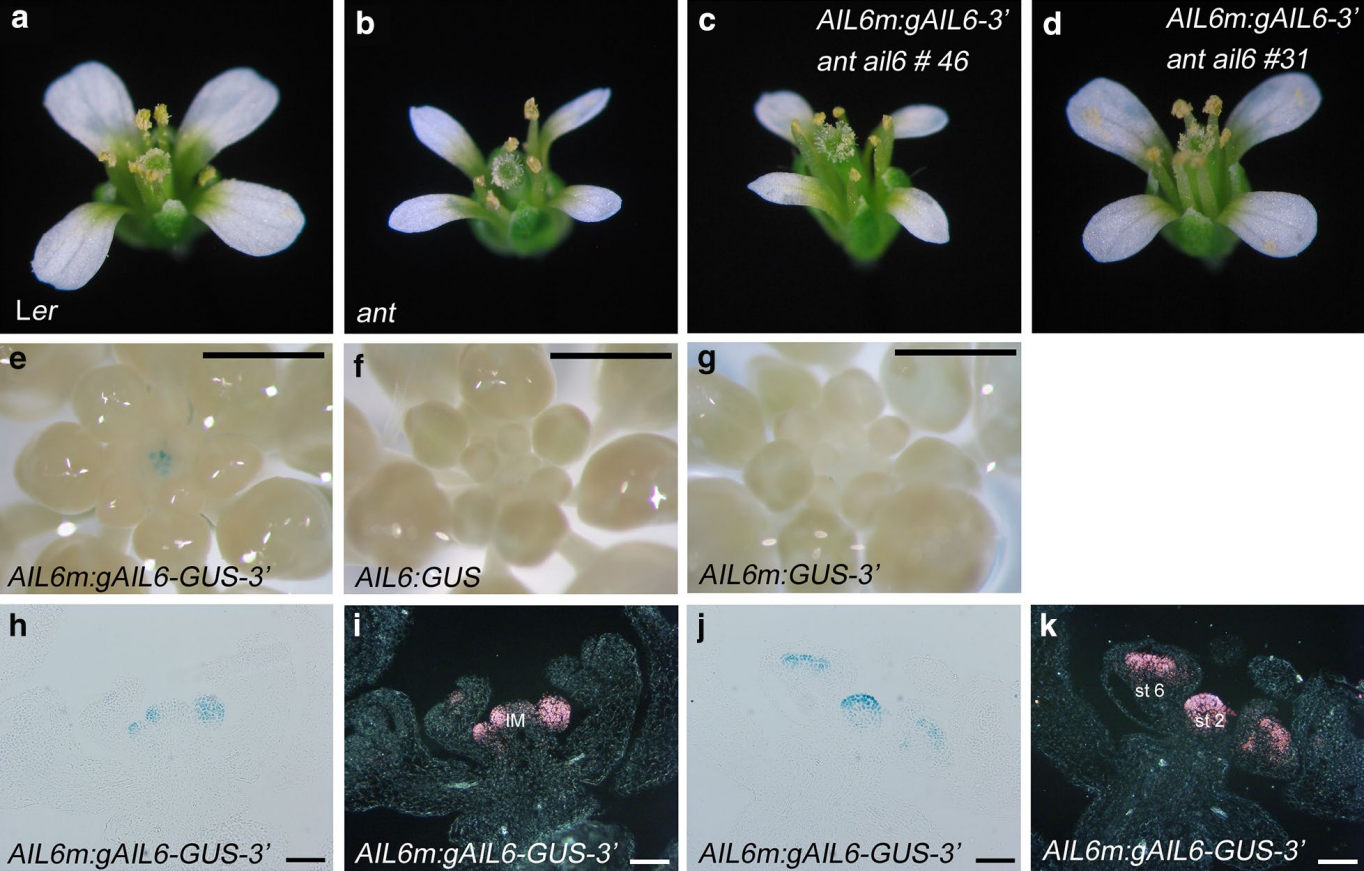
A genomic copy of *AIL6* containing 590 bp of 5′ sequence and 919 bp of 3′ sequence rescues *ant ail6* and confers normal *AIL6* expression in inflorescences. **a** L*er* flower. **b** *ant*-*4* flower. **c** *AIL6m:gAIL6*-*3′ ant ail6* *line* 46 flower. **d** *AIL6m:gAIL6*-*3′ ant ail6* *line* 31 flower. **e** GUS stained *AIL6m:gAIL6*-*GUS*-*3′* inflorescence with GUS activity detected in the young flowers in the center of the inflorescence. **f** GUS stained *AIL6:GUS* inflorescence. No staining is visible. **g** GUS stained *AIL6m:GUS*-*3′* inflorescence. No staining is visible. **h**, **i** GUS stained and sectioned *AIL6m:gAIL6*-*GUS*-*3′* inflorescence under *bright field* (**h**) and *dark field* (**i**) illumination. **j**, **k** GUS stained and sectioned *AIL6m:gAIL6*-*GUS*-*3′* inflorescence under *bright field* (**j**) and *dark field* (**k**) illumination. *Size bars* correspond to 1 mm (**e**–**g**) and 50 μm (**h**–**k**). *IM* inflorescence meristem, *st 2* stage 2 flower and *st 5* stage 5 flower

To refine the amount of 5′ sequence required for *AIL6* function, *ant*-*4/*+ *ail6*-*2* plants were transformed with a smaller *AIL6* genomic fragment containing 590 bp of 5′ sequence and 919 bp of 3′ sequence (*AIL6m:gAIL6*-*3′*) (Fig. 1a). Of the 14 transgenic lines obtained, six lines conferred partial rescue of *ant*-*4 ail6*-*2*, five lines conferred a full rescue of *ant*-*4 ail6*-*2* to the *ant*-*4* phenotype, and four lines had an over-rescue phenotype with larger petals and some stamen anthers with four locules. This range of phenotypes closely parallels that of *AIL6:gAIL6*-*3′* lines and once again is likely a consequence of variation in transgene insertion sites. Flowers for one rescued line, *AIL6m:gAIL6*-*3′ ant*-*4 ail6*-*2* line 46, and one over-rescue line, *AIL6m:gAIL6*-*3′ ant*-*4 ail6*-*2* line 31, are shown in Fig. 4a–d. Floral organ counts for *AIL6m:gAIL6*-*3′ ant*-*4 ail6*-*2* line 46 are similar to *ant*-*4* and those for *AIL6m:gAIL6*-*3′ ant*-*4 ail6*-*2* line 31 approach the numbers of floral organs in wild-type flowers (Table 1). These results indicate that 590 bp of *AIL6* 5′ region is sufficient for rescue of *AIL6* in the *ant*-*4 ail6*-*2* double mutant background.

To investigate whether this amount of *AIL6* 5′ sequence was sufficient for normal *AIL6* expression in flowers, the gene encoding the reporter β-glucuronidase (GUS) was fused in frame to *AIL6* in the *AIL6m:gAIL6*-*3′* context (i.e. *AIL6m:gAIL6*-*GUS*-*3′*; Fig. 1b) was made. Of 16 transgenic lines, GUS activity was detected in young flowers of seven lines while no staining was observed in nine lines. A representative line (*AIL6m:gAIL6*-*GUS*-*3′* line 15) was chosen for further characterization (Fig. 4e). Examination of GUS stained and sectioned *AIL6m:gAIL6*-*GUS*-*3′* line 15 inflorescences under bright and dark-field illumination shows GUS activity in a pattern that matches *AIL6* in situ hybridization data (Fig. 4h–k). GUS activity is detected throughout stage 1 and 2 flower primordia, becoming restricted to stamen and carpel primordia in stage 4 and 5 flowers (Fig. 4h–k). These results show that this *AIL6* genomic fragment is sufficient to confer a normal pattern of *AIL6* expression. In contrast, no GUS activity was detected in inflorescences of any of eight lines containing an *AIL6:GUS* reporter in which GUS is under the control of 7.7 kb of *AIL6* 5′ sequence (Figs. 1b, 4f). In addition, no GUS activity was detected in young flowers of nine *AIL6m:GUS*-*3′* lines in which GUS is present in the context of 590 bp of *AIL6* 5′ sequence and 919 bp of *AIL6* 3′ sequence (Figs. 1b, 4g). These results indicate that introns contain cis-regulatory elements responsible for *AIL6* expression in inflorescences.

### The third intron of *AIL6* contains enhancer elements that drive expression in young flowers

**Fig. 5:**
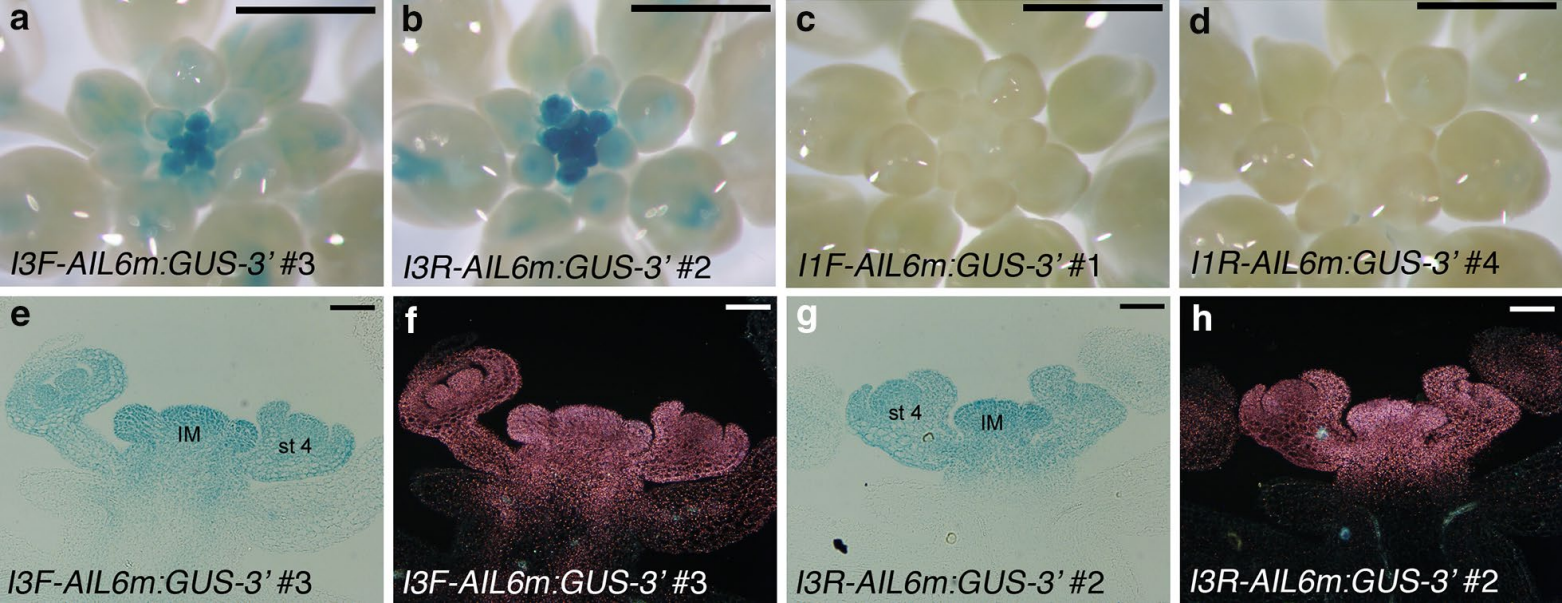
The third intron of *AIL6* confers expression throughout young flowers. **a** GUS stained *I3F*-*AIL6m:GUS*-*3′* *line* 3 inflorescence with strong GUS activity throughout the inflorescence meristem and young flowers. **b** GUS stained *I3R*-*AIL6m:GUS*-*3′* *line* 2 inflorescence with strong GUS activity throughout the inflorescence meristem and young flowers. **c**, **d** GUS stained *I1F*-*AIL6m:GUS*-*3′* *line* 1 (**c**) and *I1R*-*AIL6m:GUS*-*3′* *line* 4 (**d**) inflorescences. No GUS activity was detected. **e**, **f** GUS stained and sectioned *I3F*-*AIL6m:GUS*-*3′* *line* 3 inflorescence under *bright field* (**e**) and dark field (**f**) illumination. **g**, **h** GUS stained and sectioned *I3R*-*AIL6m:GUS*-*3′* *line* 3 inflorescence under *bright field* (**g**) and *dark field* (**h**) illumination. *Size bars* correspond to 1 mm (**a**–**d**) and 50 μm (**e**–**h**). *IM* inflorescence meristem and *st 4* stage 4 flower

To begin to map intronic sequences responsible for *AIL6* expression in inflorescences, additional GUS reporters were made in which either intron three or intron one was placed upstream of 590 bp of *AIL6* 5′ sequence (Fig. 1b). These constructs also contain 919 bp of *AIL6* 3′ sequence. Introns three and one correspond to the largest and second largest introns, respectively, within *AIL6*. GUS staining of inflorescences from the intron three constructs (i.e. *I3F*-*AIL6m:GUS*-*3′* and *I3R*-*AIL6m:GUS*-*3′*; Fig. 1b) showed staining throughout the inflorescence meristem and young flowers in a broader pattern than that observed in *AIL6m:gAIL6*-*GUS*-*3′* (compare Fig. 5a, b, e–h to Fig. 4e, h–k). In contrast, no GUS signal was observed in the young flowers of the intron one constructs (i.e. *I1F*-*AIL6m:GUS*-*3′* and *I1R*-*AIL6m:GUS*-*3′*; Figs. 1b, 5c, d). These results suggest that intron three contains enhancer elements that promote *AIL6* mRNA expression in young flowers but that additional regulatory elements are present in other regions that restrict *AIL6* expression within the inflorescence meristem and young flowers.

**Fig. 6:**
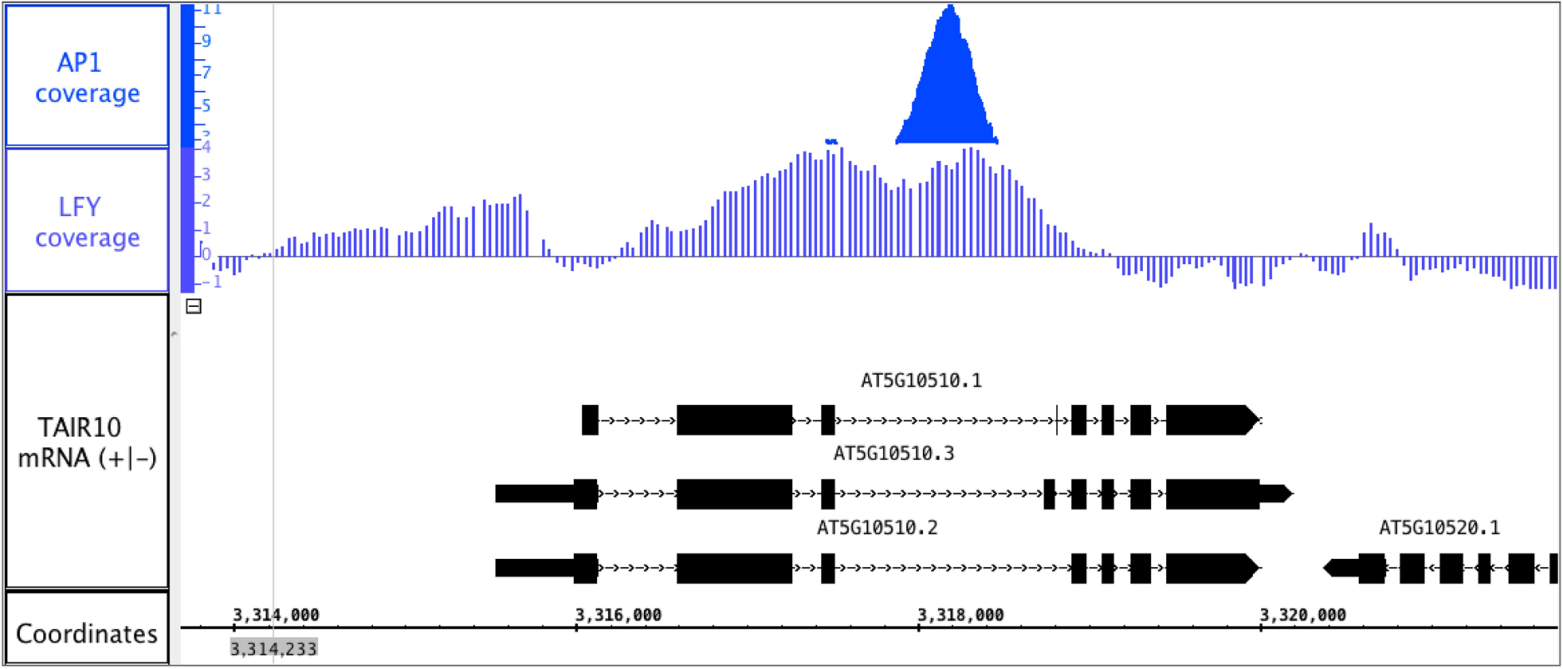
ChIP data of AP1 and LFY binding to the *AIL6* locus. Coverage graphs of ChIP-Seq (AP1) and ChIP-chip (LFY) data at the *AIL6* locus (i.e. AT5G10510) [9, 10]. The AP1 peak lies within intron three while the broad LFY peak overlaps exon two, intron two, exon three and intron three. This figure was created using Integrated Genome Browser (IGB) [16]

ChIP experiments have identified several transcription factors that appear to regulate *AIL6* expression within inflorescences. The auxin response factor MP/ARF5 promotes *AIL6* expression in groups of cells on the periphery of the inflorescence meristem to promote flower primordium initiation [8]. MP binds to several regions of *AIL6* including 5′ sequence, exon one, intron one, and intron three [8]. Two other putative regulators of *AIL6* are the floral meristem identity proteins AP1 and LFY [9, 10]. ChIP-Seq identified a binding peak for AP1 within intron three (Fig. 6) [9]. ChIP-chip identified a wide LFY binding region with two peaks that overlap exon two, intron two, exon three, and intron three (Fig. 6) [10]. These results are consistent with the identification of intron three as important in *AIL6* regulation during early stages of flower development. Further experiments will be necessary to map additional regulatory elements that in combination with intron three confer a normal *AIL6* expression pattern.

## Conclusions

This study shows the importance of intronic sequences in regulating *AIL6* transcription in flowers. Intron three of *AIL6* is sufficient to drive expression of a reporter gene in early stages of flower development. The identification of introns as important for *AIL6* regulation is consistent with ChIP data showing that several *AIL6* regulators can bind to these intronic regions. In addition, increased expression of *AIL6* is shown to partially compensate for loss of *ANT*, a gene with which *AIL6* shares some functions.

## Methods

### Plant materials and growth conditions

Plants were grown on a soil mixture of Metro-Mix 360:perlite:vermiculite (5:1:1) in 16 h days (100-150 μmol/m^2^/s) at 22 °C. *ant*-*4* was PCR genotyped as described previously [4]. *ant*-*4 ail6*-*2* double mutants, described previously [4], contain the strong *ant*-*4* allele from Landsberg *erecta* (L*er*) and *ail6*-*2* from Columbia (Col). *ant*-*4* contains a single T-to-A transversion that alters the splice site of the fourth intron, and *ail6*-*2* contains a T-DNA within the third intron.

### Plasmid construction and plant transformation

*AIL6:gAIL6*-*3′* was created by digestion of BAC F12B17 with BamHI, cloning of the resulting 12,666 bp *AIL6* genomic fragment, which contains 7729 bp of 5′ sequence and 919 bp of 3′ sequence, into pBluescript (AIL6 genomic/pBS). This *AIL6* genomic fragment was subcloned into pCGN1547. *AIL6:cAIL6*-*3′* was created by PCR amplification of *AIL6* cDNA with AIL6-27 and AIL6-22 (Table 2), digestion with KpnI and BamHI, and cloning into BJ36. The cDNA corresponds to AT5G10510.1 (Fig. 6) except that 45 nucleotides at the 5′ end of exon one, which contains an in frame upstream ATG, was included. 919 bp of *AIL6* 3′ sequence was PCR amplified with AIL6-31 and AIL6-32 (Table 2), digested with Bam and cloned into cAIL6/BJ36. A 7.7 kb 5′ fragment was made by digestion of AIL6 genomic/pBS with BamHI and AflII and addition of this fragment to a clone containing 2 kb of *AIL6* 5′ sequence (AIL6Pro2/pCRScript) to create AIL6Pro7.7/pCRScript. AIL6Pro7.7 was subsequently subcloned into the PstI site of pBluescript. AIL6Pro2 kb/pCRScript was created by PCR amplification with AIL6Pro1 and AIL6Pro8 (Table 2). For *AIL6:cAIL6*-*3′*, the 7.7 kb fragment of 5′ sequence was subcloned from AIL6Pro7.7/pBluescript into cAIL6/BJ36. For construction of *AIL6m:gAIL6*-*3′*, partial gAIL6-3′/BJ36 was constructed by digestion of AIL6 genomic/pBS with KpnI/BamHI and subcloning of the KpnI/BamHI fragment into BJ36. A 141 bp piece corresponding to the first exon of *AIL6* was PCR amplified with AIL6-27 and AIL6-1 (Table 2), digested with KpnI, and cloned into partial gAIL6-3′/BJ36 to create gAIL6-3′/BJ36. 590 bp of *AIL6* 5′ sequence directly upstream of the start codon was PCR amplified with AIL6Pro5 and AIL6Pro8 (Table 2), cloned into pCRScript and subcloned into gAIL6-3′/BJ36 cut with PstI and partially digested with NotI. *AIL6m:gAIL6*-*3′* was subcloned into pART27 using NotI. *AIL6:GUS* was constructed by subcloning of 7.7 kb *AIL6* 5′ sequence into the PstI site of pRITA. *AIL6:GUS* was subcloned into the NotI site of pMLBart. For construction of *AIL6m:gAIL6*-*GUS*-*3′*, the genomic region of *AIL6* lacking a stop codon was PCR amplified with AIL6-48 and AIL6-50 (Table 2) and cloned into the EcoRI/BamHI sites of pBluescript (gAIL6nostop/pBS). GUS was added into the BamHI site of gAIL6nostop/pBS. AIL6m was subcloned into gAIL6-GUS-3′/BJ97 cut with PstI and partially digested with NotI. *AIL6m:gAIL6*-*GUS*-*3′* was subcloned into pART27 using NotI. For construction of *AIL6m:GUS*-*3′*, AIL6m was digested from pCRScript with NotI and PstI and cloned into GUS-3′/BJ97 cut with PstI and partially digested with NotI. *AIL6m:GUS*-*3′* was subcloned into pART27 using NotI. For construction of *I3F*-*AIL6m:GUS*-*3′*, *I3R*-*AIL6m:GUS*-*3′, I1F*-*AIL6m:GUS*-*3′*, and *I1R*-*AIL6m:GUS*-*3′,* the third or first intron of *AIL6* was PCR amplified with the primers listed in Table 2 and cloned into pCRScript. The corresponding introns in the forward or reverse directions (i.e. I3F, I3R, I1F and I1R) were digested from pCRScript with NotI and EagI and cloned into *AIL6m:GUS*-*3′* partially digested with NotI. The corresponding *I3F*-*AIL6m:GUS*-*3′*, *I3R*-*AIL6m:GUS*-*3′, I1F*-*AIL6m:GUS*-*3′*, and *I1R*-*AIL6m:GUS*-*3′* were then subcloned into the NotI site of pART27. Plasmids involving PCR amplification were confirmed by sequencing. Plasmids were transformed into *Agrobacterium tumefaciens* strain ASE by electroporation. *ant*-*4/*+ *ail6*-*2* or L*er* plants were transformed with these Agrobacterium strains by vacuum infiltration [11]. Transformants were selected for kanamycin or Basta resistance.

**Table 2:** Primers used in this study

Primer name	Sequence	Purpose
AIL6-27	ATACGGTACCATGATGGCTCCGATGACGAACTGGTTAACGTTTTCTCTGTCACCAATGGAGATGTTGAGGTCATCTGA	Cloning
AIL6-22	ATTGGGATCCTTAGTAAGACTGATTAGGCCAGAG	Cloning
AIL6-31	AATAGGATCCAACCAATCATATAAGTTGATTGAG	Cloning
AIL6-32	AAGAGGATCCTCGGCTAGGAAATA	Cloning
AIL6Pro1	TAAAGTCTGCAGATAATATAGCATAGAATCATATAATATTT	Cloning
AIL6Pro8	CTGCAGAAACTTTCTTATAAAAACAATTTTAC	Cloning
AIL6-1	TTCGAGCTTTGGGATGTGAT	Cloning
AIL6Pro5	TCTCTACTGCAACTTTTGTATC	Cloning
AIL6-48	AATAGAATTCCCCGGGATGATGGCTCCGATGACGAAC	Cloning
AIL6-50	AATACTGCAGGGATCCGTAAGACTGATTAGGCCAGAG	Cloning
AIL6-52	AGTATTCGCGGCCGCGTATATATTTTCCATTCAGTTTTCG	Cloning of *I3F*-*AIL6m:GUS*-*3′*
AIL6-53	CTTACGACGGCCGTCTGTTGATTTTAAGCAGAGCC	Cloning of *I3F*-*AIL6m:GUS*-*3′*
AIL6-54	AGTATTCGCGGCCGCCTGTTGATTTTAAGCAGAGCC	Cloning of *I3R*-*AIL6m:GUS*-*3′*
AIL6-55	CTTACGACGGCCGTGTATATATTTTCCATTCAGTTTTCG	Cloning of *I3R*-*AIL6m:GUS*-*3′*
AIL6-56	AGTATTCGCGGCCGCGTACCCTTTTCTTTCTTCTTCTCT	Cloning of *I1F*-*AIL6m:GUS*-*3′*
AIL6-57	CTTACGACGGCCGTCTTTGAAAAACAAAAACAAAAAACATCAT	Cloning of *I1F*-*AIL6m:GUS*-*3′*
AIL6-58	AGTATTCGCGGCCGCCTTTGAAAAACAAAAACAAAAAACATCAT	Cloning of *I1R*-*AIL6m6m:GUS*-*3′*
AIL6-59	CTTACGACGGCCGTGTACCCTTTTCTTTCTTCTTCTCT	Cloning of *I1R*-*AIL6m:GUS*-*3′*
RTAIL6-8	GGGATAATAGCTGTAGGAGAGAAG	RT-qPCR
RTAIL6-9	TCGAGCTGCCTTATCTTCTTTG	RT-qPCR
RTFbox-1	TTTCGGCTGAGAGGTTCGAGT	RT-qPCR
RTFbox-2	GATTCCAAGACGTAAAGCAGATCAA	RT-qPCR
AIL6-FW2	AACTGGATCCTCGGAAGGACTCATCTTGCT	Cloning
AIL6-RV2	AGGTGAATTCCCCTGAACGTTGGAGTTGTT	Cloning

### RNA extraction and RT-qPCR

RNA extraction from inflorescences, cDNA synthesis and qPCR reactions were performed as described previously except that primers that do not amplify an *AIL6* transcript in the *ail6*-*2* background: RTAIL6-8 and RTAIL6-9 (Table 2) were used and in some cases RNA was extracted with Trizol with cleanup and DNase treatment performed on an RNeasy column (Qiagen) [12]. Data was normalized using AT5G15710 with the primers (RTFbox-1, RTFbox-2) shown in Table 2 [13]. The data shown are the average of two biological replicates.

### In situ hybridization

Inflorescences were fixed, embedded, sectioned, hybridized and washed as described previously except that a hybridization temperature of 53 °C was used [14]. The *AIL6* probe was made from a template corresponding to nucleotides 497–1691 of *AIL6* that was PCR amplified with AIL6-FW2 and AIL6-RV2 (Table 2) using Phusion DNA polymerase and cloned into the BamHI/EcoRI sites of pGEM3Z to create longAIL6/pGEM3Z. LongAIL6/pGEM3Z was linearized with HindIII and transcribed with T7 RNA polymerase.

### Organ counts

The first 30 flowers on five plants of each genotype were counted.

### GUS staining

The GUS assays were performed as described in [15]. The tissue was incubated in 2 mM 5-bromo-4-chloro-3-indolyl-β-glucuronic acid for 22 h. After taking pictures of whole inflorescences, the tissue was embedded in paraplast, sectioned, mounted on slides and observed under bright-field and dark-field illumination.

## References

[1] Nole-Wilson S, Tranby T, Krizek BA (2005). *AINTEGUMENTA*-like (*AIL*) genes are expressed in young tissues and may specify meristematic or division-competent states. Plant Mol Biol.

[2] Horstman A, Willemsen V, Boutilier K, Heidstra R (2014). AINTEGUMENTA-LIKE proteins: hubs in a plethora of networks. Trends Plant Sci.

[3] Galinha C, Hofhuis H, Luijten M, Willemsen V, Blilou I, Heidstra R, Scheres B (2007). PLETHORA proteins as dose-dependent master regulators of *Arabidopsis* root development. Nature.

[4] Krizek BA (2009). *AINTEGUMENTA* and *AINTEGUMENTA*-*LIKE6* act redundantly to regulate *Arabidopsis* floral growth and patterning. Plant Physiol.

[5] Mudunkothge JM, Krizek BA (2012). Three *Arabidopsis**AIL/PLT* genes act in combination to regulate shoot apical meristem function. Plant J.

[6] Prasad K, Grigg SP, Barkoulas M, Yadav RK, Sanchez-Perez GF, Pinon V, Blilou I, Hofhuis H, Dhonukshe P, Galinha C (2011). *Arabidopsis* PLETHORA transcription factors control phyllotaxis. Curr Biol.

[7] Hofhuis H, Laskowski M, Du Y, Prasad K, Grigg S, Pinon V, Scheres B (2013). Phyllotaxis and rhizotaxis in *Arabidopsis* are modified by three PLETHORA transcription factors. Curr Biol.

[8] Yamaguchi N, Wu M-F, Winter CM, Berns MC, Nole-Wilson S, Yamaguchi A, Coupland G, Krizek BA, Wagner D (2013). A molecular framework for auxin-mediated initiation of flower primordia. Dev Cell.

[9] Kaufmann K, Wellmer F, Muino JM, Ferrier T, Wuest SE, Kumar V, Serrano-Mislata A, Madueno F, Krajewski P, Meyerowitz EM (2010). Orchestration of floral initiation by APETALA1. Science.

[10] Winter CM, Austin RS, Blanvillain-Baufumé S, Reback MA, Monniaux M, Wu M-F, Sang Y, Yamaguchi A, Yamaguchi N, Parker JE (2011). LEAFY target genes reveal floral regulatory logic, *cis* motifs, and a link to biotic stimulus response. Dev Cell.

[11] Bechtold N, Ellis J, Pelletier G (1993). In planta *Agrobacterium* mediated gene transfer by infiltration of adult *Arabidopsis thaliana* plants. CR Acad Sci Ser III Sci Vie.

[12] Krizek BA, Eaddy M (2012). *AINTEGUMENTA*-*LIKE6* regulates cellular differentiation in flowers. Plant Mol Biol.

[13] Czechowski T, Stitt M, Altmann T, Udvardi MK, Scheible W-R (2005). Genome-wide identification and testing of superior reference genes for transcript normalization in *Arabidopsis*. Plant Physiol.

[14] Krizek BA (1999). Ectopic expression of *AINTEGUMENTA* in *Arabidopsis* plants results in increased growth of floral organs. Dev Genet.

[15] Mudunkothge JM, Krizek BA. The GUS reporter system in flower development studies. In: Riechmann JL, Wellmer F, editors. Methods in molecular biology: flower development methods and protocols, vol. 1110. New York: Springer; 2014. p. 295–304. 10.1007/978-1-4614-9408-9_15 24395264

[16] Nicol JW, Helt GA, Blanchard SGJ, Raja A, Loraine AE (2009). The Integrated Genome Browser: free software for distribution and exploration of genome-scale datasets. Bioinformatics.

